# Extended 2D myotube culture recapitulates postnatal fibre type plasticity

**DOI:** 10.1186/s12860-015-0069-1

**Published:** 2015-09-17

**Authors:** Sujith Sebastian, Leah Goulding, Suresh V. Kuchipudi, Kin-Chow Chang

**Affiliations:** School of Veterinary Medicine and Science, University of Nottingham, Sutton Bonington, LE12 5RD UK

**Keywords:** Postnatal, Myosin heavy chain, Differentiation, Fusion, Contraction, Fibre type switching, Porcine, Myotubes, Six1, lincRNA, Linc-MYH, Coordinated expression, Fast glycolytic, Oxidative

## Abstract

**Background:**

The traditional problems of performing skeletal muscle cell cultures derived from mammalian or avian species are limited myotube differentiation, and transient myotube persistence which greatly restricts the ability of myotubes to undergo phenotypic maturation. We report here on a major technical breakthrough in the establishment of a simple and effective method of extended porcine myotube cultures (beyond 50 days) in two-dimension (2D) that recapitulates key features of postnatal fibre types.

**Results:**

Primary porcine muscle satellite cells (myoblasts) were isolated from the *longissimus dorsi* of 4 to 6 weeks old pigs for 2D cultures to optimise myotube formation, improve surface adherence and characterise myotube maturation. Over 95 % of isolated cells were myoblasts as evidenced by the expression of Pax3 and Pax7. Our relatively simple approach, based on modifications of existing surface coating reagents (Maxgel), and of proliferation and differentiation (Ultroser G) media, typically achieved by 5 days of differentiation fusion index of around 80 % manifested in an abundance of discrete myosin heavy chain (MyHC) slow and fast myotubes. There was little deterioration in myotube viability over 50 days, and the efficiency of myotube formation was maintained over seven myoblast passages. Regular spontaneous contractions of myotubes were frequently observed throughout culture. Myotubes in extended cultures were able to undergo phenotypic adaptation in response to different culture media, including the adoption of a dominant postnatal phenotype of fast-glycolytic *MyHC 2x* and *2b* expression by about day 20 of differentiation. Furthermore, fast-glycolytic myotubes coincided with enhanced expression of the putative porcine long intergenic non-coding RNA (*linc-MYH*), which has recently been shown to be a key coordinator of *MyHC 2b* expression *in vivo*.

**Conclusions:**

Our revised culture protocol allows the efficient differentiation and fusion of porcine myoblasts into myotubes and their prolonged adherence to the culture surface. Furthermore, we are able to recapitulate in 2D the maturation process of myotubes to resemble postnatal fibre types which represent a major technical advance in opening access to the *in vitro* study of coordinated postnatal muscle gene expression.

**Electronic supplementary material:**

The online version of this article (doi:10.1186/s12860-015-0069-1) contains supplementary material, which is available to authorized users.

## Background

Skeletal muscle satellite cells as progenitor cells of multinucleated muscle fibres are one of the earliest recognised cells with stem cell-like properties [1]. Isolated satellite cells in culture are activated to undergo rapid proliferation as myoblasts which then differentiate and fuse to form post-mitotic myotubes as early muscle fibres. Muscle fibres *in vivo* are highly adapted to undergo phenotypic changes in size (hypertrophy or atrophy) and metabolic capacity (ranging from highly oxidative to enhanced glycolytic fibre type) to meet changes in physiological demands or in response to disease [2]. Newly formed myotubes undergo a maturation process of hypertrophy and metabolic remodelling during which the initial dominant embryonic and perinatal (neonatal) myosin heavy chain (MyHC) isoforms are replaced with the four main postnatal MyHC isoforms of slow, 2A, 2X and 2B, each sequentially corresponding to a fibre type of increasing glycolytic and decreasing oxidative capacity.

Although muscle satellite cells of different host species have been used in adherent two-dimension (2D) cultures for many years, to date such cultures can primarily only recapitulate the early stages of myogenesis and myotube formation [3–5]. Myoblasts often show limited efficiency of differentiation and fusion. Furthermore, as myotubes in cultures are prone to rapid loss, presumably through spontaneous contractions, their replacement by more newly-formed myotubes perpetuates an immature phenotype in culture [6]. As a consequence to such technical limitations, muscle culture experiments are typically performed on immature myotubes over a narrow window of 3 to 7 days of differentiation [3, 7]. A variety of culture media and extracellular matrices, including the use of electrospun polycaprolactone polymer coating [8], have been reported to facilitate, with limited success, myoblast differentiation and fusion, and myotube attachment. Other attempts to extend the transient persistence of myotubes included the use of three-dimension (3D) cultures of murine C2C12 muscle cells on silicon wafers [9], rat myoblasts in cantilever arrays [10], and primary rabbit muscle cells on gelatin microbeads in suspension that allowed prolonged myotube adherence and fibre maturation for up to 5 weeks with the expression of adult fast MyHCs (2A, 2X and/or 2B isoform) [6]. The use of 3D collagen mould in a chamber slide also improved primary rat myotube formation and reduced loss over a 3-week period [11]. However, such culture methods and other similar approaches have limited practicalities requiring specialised culture platforms with reduced flexibility to conduct routine cellular manipulations.

We report on a major technical breakthrough in the long term culture of myotubes. We developed a simple and highly reproducible method for the extended 2D culture of myotubes based on the strategic use of primary porcine myoblasts; the pig is an excellent model species, owing to its physiological similarity to human and relative availability, and its own importance as target species. Our method, based on modified use of surface coating reagents (Maxgel), and of proliferation and differentiation (Ultroser G) media, allowed efficient differentiation and fusion of myoblasts into myotubes that remained adherent to the culture surface for over 7 weeks of differentiation. To our knowledge, we are able for the first time to recapitulate *in vitro* the maturation process of myotubes in 2D to resemble postnatal fibre types which is a major technical advance in the ability to study phenotype plasticity.

## Methods

### Culture of porcine myoblasts and myotubes

Porcine muscle satellite cells (myoblasts) were isolated from skeletal muscles (*longissimus dorsi*) of 4 to 6 weeks old commercial Large White-cross pigs as previously described [12]. This work was approved by the School of Veterinary Medicine and Science ethical committee. Pigs were humanely euthanased according to Schedule 1 to the Animals (Scientific Procedures) Act 1986. All myoblasts and myotubes were grown on optimised coated surfaces. Into each well of a 12-well plate was applied 400 μl of 0.22 μm filter sterilised Maxgel coating mixture (MC+), comprising a 1:1 mix ratio of 1 % Maxgel ECM solution (Sigma-Aldrich, E0282-1ML; 1 in a 100 dilution with Dulbecco’s modified Eagle’s medium [DMEM]) and 2 % rat type I collagen solution (Sigma Aldrich, C3867; 1 ml rat collagen solution in 49 ml phosphate buffered saline [PBS]), which was left to fully dry overnight in a cell culture cabinet and rinsed with PBS before use. For other size plates or flasks, the volume of MC+ used was proportionally scaled.

Newly harvested satellite cells were grown in proliferation medium (PM), comprising SKGM-2 medium (Lonza, CC-3245) with added 10 % heat inactivated fetal calf serum (FCS) (Invitrogen, 10500–064), 2 % chick embryo extract (EGG Tech, 60650) and 1 % penicillin-streptomycin (P/S) (Invitrogen 15140-122), in a 37 °C incubator with gas mixture of 5 % CO2 and 5 % O2, with complete replacement of PM every 2 days. Myoblasts were passaged once at a ratio of 1:3 in PM before freezing in a mixture of 50 % FCS, 10 % dimethylsulphoxide and 40 % PM, and storing in liquid nitrogen. Depending on seeding density of thawed myoblasts, 0.5 million cells in a T75 flask should reach 60 to 70 % confluence by 3-4 days of culture.

At around 80 to 90 % confluence, the cells were rinsed and replaced with differentiation medium 1 (DM1) comprising DMEM high glucose (Invitrogen, 41965–039) with 0.4 % Ultroser G (Pall Corporation, 15950–017) and 1 % P/S. Ultroser G is a proprietary serum replacement containing a cocktail of undisclosed growth factors. Extensive myotubes should form by 3 to 4 days of differentiation. For long-term maintenance of the myotubes, from day 5–7 of differentiation, 25 % volume of the original DM1 was replaced every third day with fresh DM1, or differentiation medium 2 (DM2, DMEM high glucose with 2 % horse serum [Gibco, 26050-088] and 1 % P/S). In this way, good myotube integrity was readily maintained for several weeks.

### Cell viability

Measurement of mitochondrial activity in myotubes, as an indication of cell viability, was performed in a 96-well format with a CellTiter 96 AQueous Non-Radioactive Cell Proliferation Assay (MTS) kit (Promega) according to the manufacturer’s instructions. The MTS kit is composed of solutions of a novel tetrazolium compound [3-(4,5-dimethylthiazol-2-yl)-5-(3-carboxymethoxyphenyl)-2-(4-sulfophenyl)-2H-tetrazolium, inner salt; MTS] and an electron coupling reagent (phenazine methosulfate). MTS is bioreduced by cells into a formazan product that is soluble in cell culture medium. The conversion of MTS (measured at 490 nm absorbance) into the aqueous soluble formazan product is accomplished by dehydrogenase enzymes found in metabolically active cells. The quantity of formazan product is directly proportional to the number or activity of living cells in culture.

### Real time PCR

RNA extraction from myoblasts and myotubes was performed with an RNeasy fibrous tissue mini kit (Qiagen). TaqMan real-time PCR was used to quantify the expression of six porcine MyHC gene isoforms (*MyHC embryonic*, *MyHC perinatal*, *MyHC slow/I*, *MyHC 2a*, *MyHC2x* and *MyHC 2b*) using primers and TaqMan probes as previously described [13–15]. Forward and reverse primers for the SYBR Green detection of putative porcine *long intergenic non-coding* (*linc*)*-MYH* (exon 5) are 5′-GAGGCTCGGGAAGGAATCC-3′ and 5′-TGCCCTCTGGTGGTAAAAGC-3′. Forward and reverse primers for porcine *Six1* and *Eya1* detection are 5′-GTTCAAGAACCGAAGGCAAC-3′ and 5′-CCCCTTCCAGAGGAGAGAGT-3′, and 5′-CAGCTCTCCATATCCAGCACATT-3′ and 5′-TTTGTGGACGGCGTCGTA-3′ respectively. A relative standard curve was used to quantify the expression of each gene normalised to its corresponding 18S rRNA expression.

## Results

### Culture of primary porcine muscle cells

**Fig. 1:**
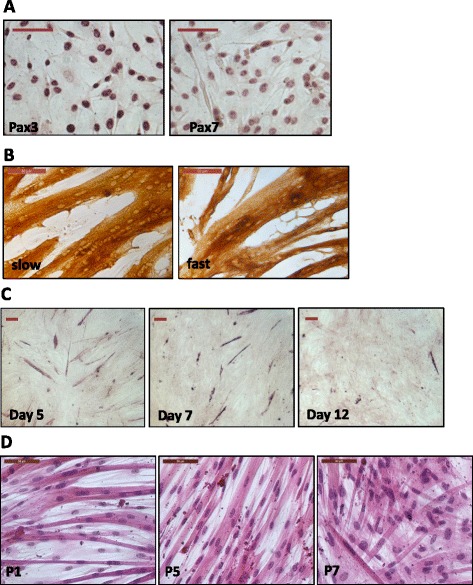
Characterisation of primary porcine muscle cells. **a** Highly enriched myoblasts were isolated from 4 to 6 week old muscles (*longissimus dorsi*), as evidenced by nuclear detection of Pax3 and Pax7 (both antibodies from R&D Systems, 1:100 dilution). **b** Extensive discrete immunodetection of MyHC slow (Sigma, M8421, 1:4000 dilution) and fast (Sigma, M4276, 1:400 dilution) in myotubes at day 5 differentiation. **c** By contrast, myotube formation was typically low using standard media (in this case MEM with 10 % fetal bovine serum and 1 % penicillin-streptomycin (P/S) as proliferation medium, and MEM with 2 % horse serum and 1 % P/S as differentiation medium [12, 19]). Immunodetection of α-actin was confined to a limited number of myoblasts/myotubes over 12 days of differentiation. Day = days of differentiation. **d** Present protocol conferred comparably high levels of myotube formation over 7 passages (P) of porcine myoblasts. Hematoxylin and eosin (H&E) stained myotubes at day 3 differentiation from P1, P5 and P7

Greater than 95 % of isolated porcine muscle cells, derived from the *longissimus dorsi* of 4 to 6 weeks old pigs, expressed transcription factors Pax3 and Pax7 (Fig. 1a) indicating that the isolated cells were highly enriched for muscle satellite cells [16, 17]. Porcine myoblasts were grown in PM in culture plates or flasks coated with a specified volume of MC+ that was fully dried overnight (see Methods). When myoblasts reached around 80 % confluence, usually by day 3 or 4 of culture from frozen stock, PM was replaced with differentiation medium containing Ultroser G (DM1). By 5 days of differentiation, we typically detected an abundance of discrete MyHC slow and fast myotubes (Fig. 1b) which was in marked contrast to conventional cultures for porcine myotubes where few myotubes were found (Fig. 1c). With our improved protocol, the efficiency of myotube formation remained unaffected over seven myoblast passages (Fig. 1d).

### Enhanced myotube differentiation and sustained viability

**Fig. 2:**
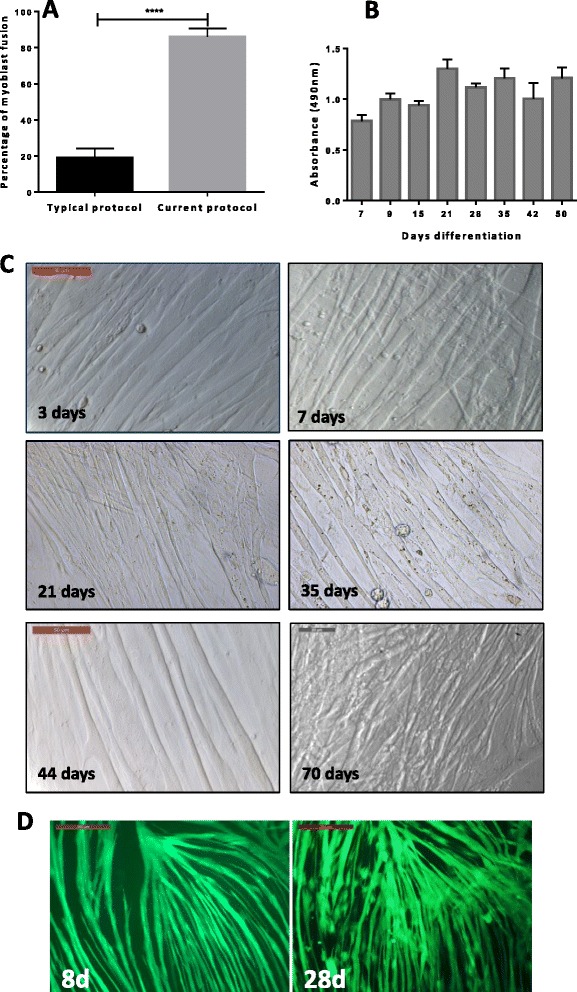
Enhanced myotube formation and viability. **a** Present culture protocol conferred highly efficient myotube formation relative to typical cultures [12, 19]. About 80 % of fusion could be typically achieved by day 5 of differentiation (error bars = standard deviation, **** = *P*≤ 0.0001 based on two-sample unpaired *t* test). **b** Myotube mitochondrial activity as determined by MTS assays remained relatively unchanged over a period of 50 days of differentiation. **c** From day 5 of differentiation, 25 % of DM1 was replaced with DM2 every third day for extended myotube maintenance to at least day 50 of differentiation (bar = 200 μm). **d** Porcine myotubes transduced with GFP-expressing non-replicating adenovirus at day 8 remained largely intact at day 28 of differentiation based on persistence of green fluorescence which was indicative of extended myotube survival in culture (bar = 200 μm)

**Fig. 3:**
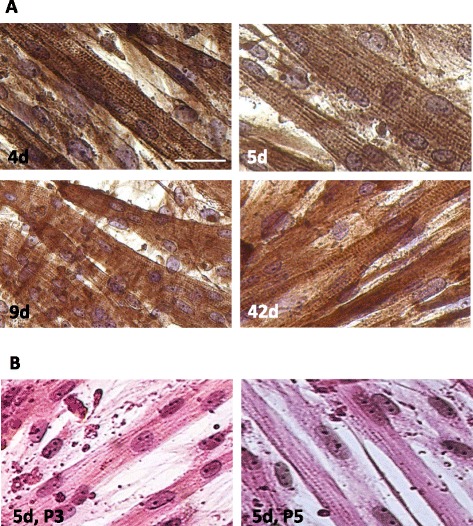
Myotube formation was accompanied by the observation of lateral striations indicative of sarcomeric structures. **a** Myotubes at 4d, 5d, 9d (DM1) and 42d (DM1) of differentiation were immunostained for the presence of sarcomeric α-actin (Sigma A2172, 1:500 dilution). **b** Sarcomeric lateral striations were visible in H&E stained myotubes differentiated for 5d, derived from P3 and P5 *longissimus dorsi* myoblasts. Bar = 50 μm for all panels

With our approach, by day 5 of differentiation, fusion index was typically around 80 % (Fig. 2a). To maintain myotube integrity over several weeks, from day 5 to 7 of differentiation, a quarter of DM1 was replaced with fresh DM1 or DM2 (with horse serum) at every third day of culture. There was little deterioration in myotube viability based on mitochondrial activity (MTA assays) over 50 days of differentiation, and morphological appearance (Fig. 2b and c). Myotubes transduced at day 8 with replication-defective adenovirus expressing green fluorescence protein (GFP) were found to persist in culture for at least 28 days of differentiation which demonstrated that the culture conditions applied were conducive to extended myotube survival, a requisite for myotube maturation (Fig. 2d). Formed myotubes throughout the differentiation period manifestly possessed lateral striations characteristic of sarcomeres which remained visible throughout several rounds of myoblast passages (Fig. 3).

### Extended myotube culture displayed phenotypic plasticity and postnatal phenotype

**Fig. 4:**
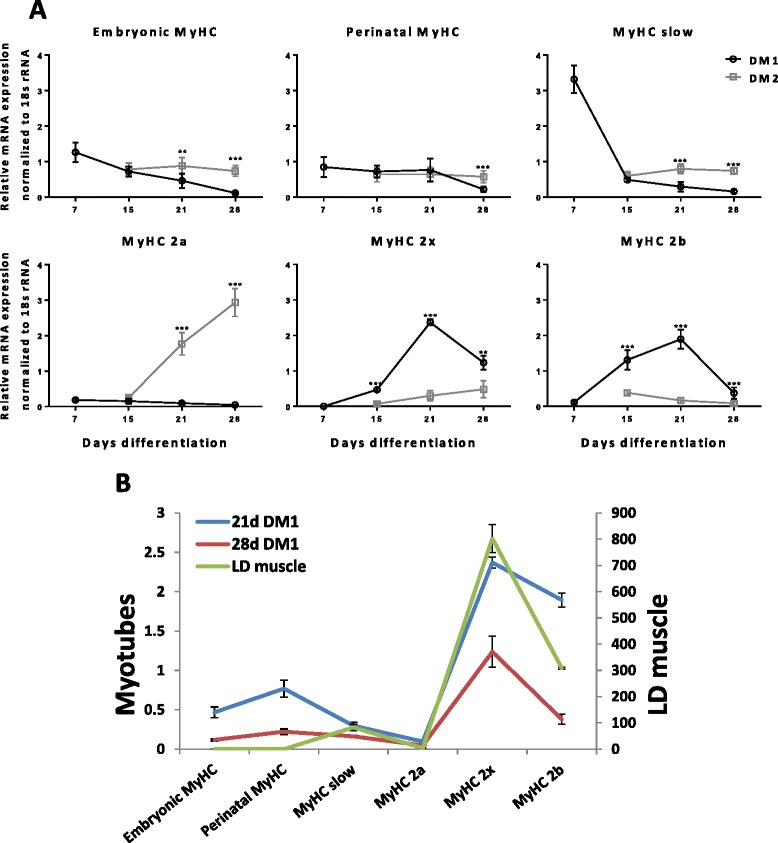
Extended myotube cultures exhibited phenotype plasticity and postnatal fibre phenotype. **a** Full complement of pre- and post-natal *MyHC* RNA isoforms in myotubes at 7, 15, 21 and 28 days of differentiation were quantified by real-time PCR (normalised to 18S rRNA). Use of DM1 (with Ultroser G) and DM2 (with horse serum) resulted in divergent *MyHC* profiles from day 15 of differentiation. *MyHC* profiles shown are a representative set of three independent experiments. **b** The use of DM1, but not DM2, promoted myotube transition towards a fast-glycolytic *MyHC* profile that resembled postnatal *longissimus dorsi* (LD) muscle of a 22-week-old Duroc pig. Error bars = standard deviation; ** = *P* ≤0.01, *** = *P* ≤0.001 based on two-sample unpaired t test at indicated time points

An exciting observation made on extended myotube cultures over 28 days of differentiation was that the choice of DM could profoundly affect the profiles of *MyHC* expression. Despite absence of innervation, myotube cultures by 21 days in DM1 (with Ultroser G) underwent fast phenotypic changes of raised fast *MyHC 2x* and *2b* expression (Fig. 4a) which resembled the relative expression pattern of *MyHCs* in *longissimus dorsi* (fast) muscle of a 22 week old pig (Fig. 4b). Interestingly, testosterone was one ingredient identified by high throughput metabolomic analysis in the proprietary serum substitute Ultroser G (data not shown) but its influence on postnatal MyHC expression is unclear. The use of DM2 (with horse serum), by contrast, appeared to maintain a phenotype of sustained *MyHC embryonic* and *perinatal* expression, and preferentially up-regulated the expression of the oxidative *MyHC slow* and *2a* genes (Fig. 4a). These results demonstrated the plasticity of myotubes in being able to undergo phenotypic changes in response to different culture media. Furthermore, regular spontaneous contractions of myotubes were frequently observed throughout much of the extended culture period; contractions appeared earlier, more vigorous and extensive with the use DM1. Additional movie files (see Additional file 1, Additional file 2, Additional file 3, Additional file 4 and Additional file 5) show contracting myotubes in DM1 or DM2 at 4, 8, 9, 27 and 60 day differentiation respectively. Thus, with relatively simple but significant culture modifications, we were able to induce extensive myotube formation, and maintain myotube attachment and viability for several weeks of differentiation. These results were particularly gratifying as porcine myoblasts have been recognised to be least efficient amongst the commonly used host species (rodents, rabbit, chicken, duck and human) to differentiate into myotubes [12, 18, 19].

### Rising putative *linc-MYH* expression coincided with accumulation of *MyHC 2x* and *2b* in myotubes

**Fig. 5:**
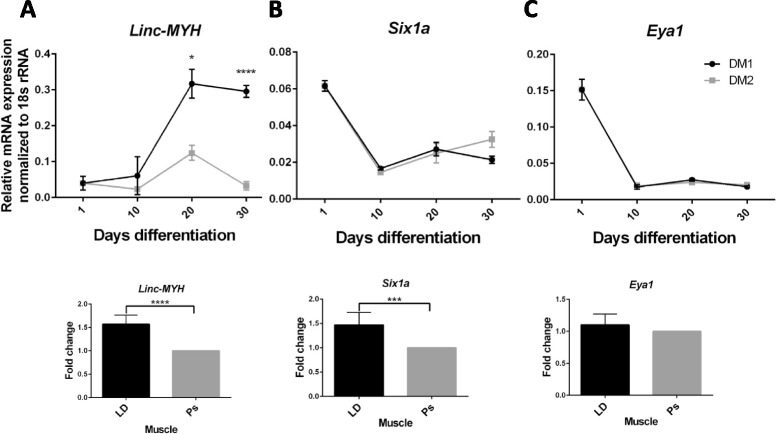
Induction of *linc-MYH* expression paralleled elevated expression of fast MyHC 2x and 2b in myotubes. The expression of *linc-MYH* (**a**), *Six1* (**b**) and *Eya1* (**c**) was determined by real-time PCR (normalised to 18S rRNA) in extended myotube cultures in DM1 (with Ultroser G) and DM2 (with horse serum), and skeletal muscles: fast *longissimus dorsi* (LD) and slow *psoas* (Ps). Skeletal muscle data shown are the combined results of three sets of LD and Ps from three 22-week-old pigs. Error bars = standard deviation; * = *P* ≤0.05, *** = *P* ≤0.001, **** = *P* ≤0.0001 based on two-sample unpaired t test at indicated time point

Expression of the long intergenic non-coding gene, *linc-MYH*, located in the 70 kb intergenic region between *Myh3* (*MyHC embryonic*) and *Myh2* (*MyHC 2a*) of the skeletal *MyHC* cluster, was recently shown to confer the coordinated adult fast-type (in particular *MyHC 2b*) and prevent the slow-type programme in murine *tibialis anterior* muscle [20]. Interestingly, the promoters of *linc-MYH*, *MyHC 2b* and *2x* are controlled in common by a Six1-dependent enhancer located at the immediate 5′-end of the *linc-MYH* gene within the murine *Myh3*-*Myh2* intergenic region [20]. Six1, a homeobox transcription factor, along with its cofactor Eya1, also activates the adult fast-twitch and represses the slow-twitch programme in muscle fibres [21, 22]. By sequence alignment (EMBOSS Matcher) of the 73 kb porcine *Myh3*-*Myh2* intergenic region with the murine *linc-MYH* RNA (2310065F04Rik), we identified all 5 putative porcine *linc-MYH* exons at modest similarities (as expected) of 58 to 67 % with corresponding murine exons [23]. We compared the expression of *linc-MYH*, *Six1* and *Eya1* in our extended myotube cultures to determine their possible involvement in fast phenotype determination (Fig. 5). Beyond 10 days of differentiation, *linc-MYH* expression was much more highly up-regulated in DM1 than DM2 (Fig. 5a) which corresponded with the much higher expression of *MyHC 2x* and *2b* in DM1 myotubes (Fig. 4a and b). *Six1* and *Eya1*, on the other hand, showed sharp reduction in expression from day 1 of differentiation in both DM1 and DM2 cultures (Fig. 5b and c). *Linc-MYH* and *Six1*, as predicted, were more highly expressed in the fast *longissimus dorsi* than slow *psoas* muscle of 22-week-old pigs (Fig. 5). Therefore, the fast postnatal MyHC phenotype in extended DM1 cultures strongly correlated with enhanced *linc-MYH* expression but not with *Six1* expression.

## Discussion

The usefulness of myotubes in cultures to examine muscle gene regulation or response to external stimuli has hitherto been limited by the immature state of myotubes derived from a range of mammalian (human, pig, rabbit, cow, horse, mouse and rat) [3, 4, 6, 7, 12, 18, 19] and avian (chicken and duck) [5, 12] species. Most published experiments on myotubes are performed within about a week of differentiation, at a stage when adult MyHCs (namely MyHC 2X and 2B) are largely lacking. The principal reasons for this predicament are limited efficiency in myotube formation and continual loss of newly formed myotubes. The ability to culture myotubes long term in 2D to resemble the postnatal phenotype is of high biomedical value. The most important biomedical or veterinary muscle conditions (such as muscle hypertrophic growth [24], muscle wasting or atrophy [25], changes in fibre type composition affecting muscle performance [26] or even meat quality [27], and obesity-related insulin resistance [28, 29]) all involve skeletal muscle in the postnatal fibre state. To have a suitable *in vitro* platform to examine such basic underlying changes is clearly advantageous. Until now such a platform has been elusive in that myotube differentiation and maturation were major limiting factors. The present paper reports on such a technical breakthrough that promotes myotube differentiation and accommodates spontaneous myotube contractions without detachment which would facilitate the *in vitro* study of all aspects of postnatal muscle fibre biology.

In the first weeks of postnatal development in rats and mice, there is typically progressive loss of embryonic and perinatal MyHCs and accumulation of MyHC 2A, 2X and 2B isoforms in designated fast fibre populations [2]. Our extended porcine myotube cultures in DM1 showed similar changes where reduction in *embryonic* and *perinatal MyHC* expression was accompanied by dominant expression of *MyHC 2x* and *2b* (Fig. 3a). Furthermore, the pattern of relative *MyHC* expression at day 21 and 28 of differentiation in DM1 showed resemblance to the *MyHC* expression profile of a 22 week old pig *longissimus dorsi* muscle (Fig. 4b) [14]. The divergence in *MyHC* profiles between DM1 and DM2 is biologically significant as it demonstrated the expression plasticity of postnatal *MyHC* genes through the use of different culture media. *In vivo*, early postnatal changes in *MyHC* gene expression that lead to the formation of adult fibre types are dependent on the establishment of corresponding fast and slow motor units, load bearing after birth, and thyroid hormone surge in the case of fast MyHC induction [2]. The ability of our myotubes to adopt fast or slow *MyHC* profile in the absence of innervation indicates that the choice of particular culture conditions is also an important phenotype determinant. DM1 myotube culture over several weeks recapitulated a fast-like postnatal pattern of *MyHC* expression. We have therefore established a 2D culture platform that is conducive to the study of coordinated gene changes that govern fibre type and associated phenotypic alterations.

Acquiring a fundamental understanding of preferential up- or down-regulation of specific MyHC isoforms *in vitro* could facilitate our ability to manipulate phenotypic changes *in vivo* for beneficial biomedical and veterinary outcomes. The present culture platform opens up a convenient controlled environment to investigate a range of mechanisms and factors that are involved in the coordinated expression of muscle gene isoforms, such as the roles of transcription factors like NFATc1 [30], microRNAs [31–33], and anti-sense [34] and linc [20] RNAs in the differential regulation of *MyHC* and other fibre type-specific genes. We can systematically interrogate the role or effectiveness of individual genes or compounds on coordinated MyHC isoform switching or myotube development. As an exemplification, we found that the fast glycolytic myotube phenotype of elevated *MyHC 2x* and *2b* expression (under DM1 culture condition) closely mirrored the rising profile of putative porcine *linc-MYH* RNA expression but not with that of *Six1* and *Eya1*. In muscle, on the other hand, the fast phenotype has been shown to correlate with the up-regulation of *linc-MYH* [20] and the presence or over-expression of Six1 [21, 22, 35]. Our finding of an inverse relationship between *linc-MYH* RNA and *Six1* expression (Fig. 5) suggests that factors other than Six1 could be responsible for the induction of *linc-MYH* RNA in growing myotubes.

Another research opportunity is to examine *in vitro* the role of thyroid hormone and other growth factors in the programming of fast phenotype to dissect the qualitative and quantitative changes of orchestrated gene expression during the transition [34, 36]. Access to largely pure cultures of myotubes of a particular phenotype would also make the isolation of myonuclei and subsequent study of chromatin modifications much simpler than the use of whole muscle tissues. Finally, extended myotube cultures would allow us to better scrutinise whether there are intrinsic differences in the conferment of myotube phenotype between satellite cells of fast and slow muscles from the same animal or between animals of different ages.

## Conclusion

In conclusion, we have made a major technical breakthrough to be able to culture well differentiated porcine myotubes in 2D over an extended period of at least 50 days. Furthermore, we showed that cultured myotubes could be made to adopt a fast adult phenotype of dominant *MyHC 2x* and *2b* expression. For the first time, to our knowledge, we are able to recapitulate the maturation process of myotubes *in vitro*, opening new opportunities to study coordinated postnatal muscle gene expression.

## Additional files

Additional file 1:
**4 day differentiation LD.avi.** Contracting myotubes in DM1 at 4 day differentiation. (AVI 6059 kb) Additional file 2:
**8 day differentiation LD.mp4.** Contracting myotubes in DM2 at 8 day differentiation. (MP4 56597 kb) Additional file 3:
**9 day differentiation LD.avi.** Contracting myotubes in DM2 at 9 day differentiation. (AVI 182623 kb) Additional file 4:
**27 day differentiation LD.mp4.** Contracting myotubes in DM1 at 27 day differentiation. (MP4 3834 kb) Additional file 5:
**60 day differentiation LD.wmv.** Contracting myotubes in DM1 at 60 day differentiation. (WMV 2609 kb)

## Abbreviations

2D: Two-dimension
MyHC: Myosin heavy chain
linc: Long intergenic non-coding
3D: Three-dimension
MC+: Maxgel coating mixture
PM: Proliferation medium
DM: Differentiation medium
GFP: Green fluorescence protein
LD: *Longissimus dorsi*
